# Conservation of genetic uniqueness of populations may increase extinction likelihood of endangered species: the case of Australian mammals

**DOI:** 10.1186/s12983-016-0163-z

**Published:** 2016-07-08

**Authors:** Andrew R. Weeks, Jakub Stoklosa, Ary A. Hoffmann

**Affiliations:** School of BioSciences, Bio21 Institute, The University of Melbourne, Parkville, VIC 3010 Australia; School of Mathematics & Statistics and Evolution & Ecology Research Centre, The University of New South Wales, Kensington, NSW 2052 Australia

**Keywords:** Threatened species, Adaptation, Genetic diversity, Evolutionary Significant unit, Extinction risk

## Abstract

**Background:**

As increasingly fragmented and isolated populations of threatened species become subjected to climate change, invasive species and other stressors, there is an urgent need to consider adaptive potential when making conservation decisions rather than focussing on past processes. In many cases, populations identified as unique and currently managed separately suffer increased risk of extinction through demographic and genetic processes. Other populations currently not at risk are likely to be on a trajectory where declines in population size and fitness soon appear inevitable.

**Results:**

Using datasets from natural Australian mammal populations, we show that drift processes are likely to be driving uniqueness in populations of many threatened species as a result of small population size and fragmentation. Conserving and managing such remnant populations separately will therefore often decrease their adaptive potential and increase species extinction risk.

**Conclusions:**

These results highlight the need for a paradigm shift in conservation biology practise; strategies need to focus on the preservation of genetic diversity at the species level, rather than population, subspecies or evolutionary significant unit. The introduction of new genetic variants into populations through in situ translocation needs to be considered more broadly in conservation programs as a way of decreasing extinction risk by increasing neutral genetic diversity which may increase the adaptive potential of populations if adaptive variation is also increased.

**Electronic supplementary material:**

The online version of this article (doi:10.1186/s12983-016-0163-z) contains supplementary material, which is available to authorized users.

## Background

Defining significant species and populations for the purpose of biological conservation can be fraught with problems. There are over 26 separate definitions of species hindering conservation efforts [1], with some definitions leading to a 50 % increase in currently recognised species [2], yet none able to solve the apparent species ambiguity problem [3]. Within species, importance is often given to “unique” populations, labelled as subspecies, chromosomal races, morphospecies, ecotypes and so on (e.g., [4–8]). The biological importance of these populations is not always clearly understood, and can be based on geographical/political boundaries (e.g., the subspecies status of the American puma, *Puma concolor*), morphology, ecology, genetics and/or a mix of the above [5, 7, 9–11].

In species of conservation concern, the Evolutionarily Significant Unit (ESU) was proposed to identify unique population(s) that have evolved independently for a long period of time, are genetically differentiated and uniquely adapted to their environment [11, 12]. By identifying ESUs, it was argued that managers could prioritise conservation efforts so that at the very least, unique populations would be preserved, thereby maintaining the processes that lead to adaptive differentiation [13]. The advent of molecular techniques, however, led to ESUs being largely defined by neutral genetic markers (e.g., monophyly at mitochondrial markers [10]), with further delineation of populations into Management Units (MUs) depending on the degree of differentiation at nuclear loci [10, 13]. This provided a routine way of characterising unique populations, and has gained widespread use in conservation despite the focus being on relatively few neutral genetic markers.

While there is often acknowledgement that adaptation and ecological diversity should also be considered when defining ESUs (and MUs), as originally defined by Ryder [11], frequently ESUs and MUs are defined entirely through such marker systems (e.g., [14–19]). For threatened species programs, this creates a challenge because conservation efforts are targeting populations rather than the species more generally, and thereby potentially promoting fragmentation. This strategy might make sense if past unique evolutionary trajectories in populations are being conserved; but what if these populations aren’t really “unique” at all, and therefore being managed in such a way that may increase their risk of extinction due to reductions in genetic diversity and loss of population fitness?

Random genetic drift effects will affect small populations greater than larger populations, and we therefore hypothesise that in many threatened species there will be a relationship between the level of genetic diversity and population “uniqueness”. We show using neutral marker genetic datasets from five Australian mammals that random genetic drift (and not mutation) is likely to be responsible for the uniqueness of populations of many threatened taxa; by managing such populations as subspecies, ESUs and/or MUs, the extinction risk of the entire species is likely to be increased based on genetic grounds. Introducing new genetic variants into populations through translocation is likely to benefit populations that have undergone declines in genetic diversity through strictly drift processes. While ideally ecological diversity and adaptive genetic potential should also be considered when conserving populations, conservation decisions in most threatened species programs currently remain based on relatively few neutral markers, and we suggest a novel method for identifying unique populations based on such data.

## Methods

### Species and microsatellite datasets

We analysed microsatellite datasets from previous studies [20–23] for five threatened species within Australia that have highly fragmented populations; the mountain pygmy possum (*Burramys parvus*), the eastern barred bandicoot (*Perameles gunnii*), the eastern quoll (*Dasyurus viverrinus*), the northern quoll (*Dasyurus hallucatus*) and the tiger quoll (*Dasyurus maculatus*). All species are listed under the Australian Environment Protection and Biodiversity Conservation (EPBC) Act 1999 or International Union for Conservation of Nature (IUCN), are considered to be in a state of decline, and consist of populations that have been given the status of subspecies (*D. maculatus*, *P. gunnii*), ESUs (*B. parvus*, *D. hallucatus*, *D. maculatus*) and MUs (*B. parvus*, *D. hallucatus*, *D. maculatus*, *D. viverrinus*) (Table 1).

**Table 1:** Threatened status, previously recognised population uniqueness, and microsatellite information for each species in this study

				Population uniqueness			Microsatellite datasets			
Species	EPBC Act Status^a^	IUCN Status^b^	Population trend^a^	Sub species	ESUs	MUs	No. Populations^c^	No. Individuals	No. Loci	Reference
*Burramys parvus*	endangered	critically endangered	declining	-	3	Yes	12	762	8	[22]
*Perameles gunnii*	vulnerable	nr. threatened	declining	2	NA	NA	9	286	12	[23]; Weeks unpubl. data
*Dasyurus viverrinus*	not listed	nr. threatened	declining	-	-	Yes	10	425	7	[20]
*Dasyurus hallucatus*	endangered	endangered	declining	4	2	Yes	7	172	6	[20]
*Dasyurus maculatus*	endangered	endangered	declining	2	2	Yes	12	450	6	[20]

*NA* no study has been undertaken to determine ESU or MU status

^a^Taken from the Australian Federal Government website for threatened species (see http://www.environment.gov.au/)

^b^Taken from the IUCN red list (http://www.iucnredlist.org)

^c^For *D. hallucatus*, only the most contemporary (2006) samples were used in analyses from sites where multiple samples were taken through time

*Burramys parvus* is considered endangered under the EPBC Act 1999, has a highly fragmented distribution confined to the alpine and sub-alpine zones of Australia, with populations split into three ESUs [22]. *Dasyurus hallucatus* is also endangered under the EPBC Act 1999, has previously been split into four subspecies based on morphology [24], but is now recognised as consisting of two ESUs and several MUs [25]. *Dasyurus maculatus* is currently split into two subspecies and two ESUs that are not concordant [26], with the species listed as endangered under the EPBC Act 1999. *Dasyurus viverrinus* is not listed under the EPBC Act 1999 but is in decline, with the species consisting of a number of MUs on the island of Tasmania and thought to be extinct on the mainland of Australia [21]. *Perameles gunnii* is split into two subspecies, one found on the mainland of Australia and listed as endangered under the EPBC Act 1999, the other found on the island of Tasmania and listed as vulnerable [23].

Populations and sample sizes are those reported for *B. parvus*, *D. viverrinus*, *D. maculatus*, and *D. hallucatus* (see Table 1), except populations with samples sizes < 15 individuals were removed from analyses. For *P. gunnii*, analysed contemporary samples came from six sites in Tasmania and three sites in Victoria, Australia and genotyped at 12 loci. Published and accessible datasets used in this study have been deposited on Dryad.

For each dataset, mean expected heterozygosity (*H*_e_) and allelic richness over loci (*A*_r_) were calculated as measures of genetic diversity in FSTAT [27] for observed data. These were regressed against the mean population-specific *F*_ST_ estimates for each population (genetic uniqueness) calculated in GESTE [28]. In addition to the population-specific *F*_ST_, we also regressed the measures of genetic diversity against the mean pairwise *F*_ST_ for each population (based on each pairwise comparison for populations within a species). This always gave a similar, but slightly weaker relationship (data not presented).

### Terrestrial mammals listed under the Australian EPBC Act 1999

There are 86 terrestrial mammals listed under the Australian EPBC Act 1999 (30 October 2015) as critically endangered (6), endangered (31) or vulnerable (49), with two already presumed extinct [29]. The listed mammals include species, subspecies and unique populations (see http://www.environment.gov.au), although there is likely to be taxonomic uncertainty in many cases [29]. We used this list to gain further insight into the extent of the relationship between genetic diversity and uniqueness and highlight the impact on management decisions. For listed species we searched the literature for (a) evidence of fragmentation, and (b) genetic data reporting population based estimates of genetic variation (allelic richness) and pairwise *F*_ST_. Where data was available, we then regressed genetic diversity (allelic richness) against mean pairwise *F*_ST_ for each population, as above.

## Results

**Fig. 1:**
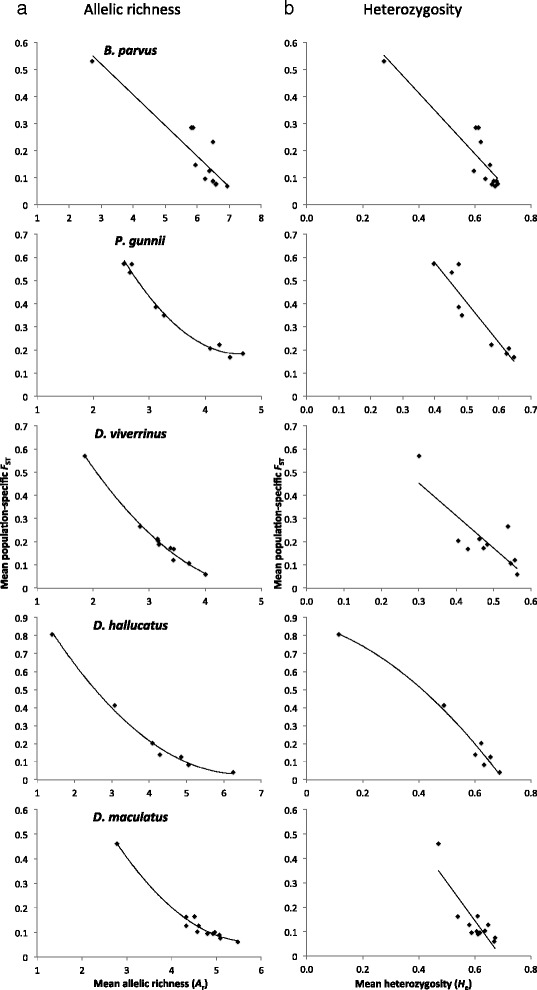
Regressions between mean population-specific *F*_ST_ and genetic diversity. Mean allelic richness (**a**) or mean heterozygosity (**b**) regressed against mean populations-specific *F*_ST_ for five threatened Australian mammal species with estimates based on nuclear microsatellite loci. Best-fit regression curves are indicated (see Additional file 1)

Using the approach in Coleman et al. [30] and consistent with expectations [31], we found a highly significant negative relationship (linear or quadratic) between both genetic diversity measures (*H*_e_ and *A*_r_) and genetic uniqueness (population-specific *F*_ST_) for each dataset (Fig. 1; Additional file 1). Therefore, the more unique a population, the lower the level of neutral genetic variation present within that population. We hypothesised that this relationship is likely to be driven by random genetic drift processes and small effective population size (leading to loss of genetic diversity and changes in allele frequencies). On the other hand, mutation-driven divergence without much loss of genetic diversity could reflect long term adaptive evolutionary change, if it is assumed that mutation-driven divergence in microsatellites also reflects mutation-driven changes in functional genes. We explored this using simulations combining observed microsatellite data from populations for each species and found a consistent and highly significant negative relationship between population-specific *F*_ST_ and genetic diversity (see Additional file 1), as expected by theory [31]. Therefore, the strongly negative relationship found in observed data for each species and shape of this relationship is likely to be largely explained by drift processes.

The tight relationship found between *A*_r_ and population-specific *F*_ST_ under a drift model provides a way of defining unique populations, even in the presence of strong drift effects. This negative relationship is expected by theory [31] and the simulations (Additional file 1) with real datasets confirm this expectation. However, populations that sit above the 95 % prediction intervals (and therefore more likely to be genetically differentiated than expected purely by drift) are likely to have unique mutations that have accumulated within those populations through time. We tested this by undertaking the same simulation for a large population (*N* = 5000) for 5000 generations, where we would expect mutation to introduce more novel alleles. For each species, the simulated population sits well above the 95 % prediction intervals for *A*_r_ (Additional file 1), indicating genetic uniqueness (differentiation) higher than that expected by drift effects alone. *H*_e_ generally sits within the 95 % prediction intervals, but this is not surprising, as novel alleles will not necessarily increase heterozygosity. As well as the microsatellite loci having a higher number of novel mutations, there is also an increased likelihood of novel mutations at functional loci, raising the issue of whether these more differentiated populations are adaptively diverging and now heading down different evolutionary trajectories.

Of the 84 subsisting terrestrial mammals listed under the Australian EPBC Act 1999, there is a total of 73 different species listed, with 31 (37 %) identified as subspecies and/or significant populations (Additional file 1). Of these 73 species, there is evidence of 78 % (57) having heavily fragmented and declining populations, 12 % (6) being unfragmented or only present as a single small population, and 10 % (16) for which information is lacking (Additional file 1). Population based genetic data (either of mitochondrial or nuclear origin) exists for 65 % (33) of listed mammal species, with published microsatellite data known for 38 % (28). Of the 28 species where microsatellite data has been published, population based estimates of *A*_r_ and *F*_ST_ have been reported for 15 species for greater than three populations, allowing us to test for a relationship between genetic diversity and genetic uniqueness (Table 2). We found a significant negative relationship between genetic diversity (*A*_r_) and genetic uniqueness (population *F*_ST_) in 11 of the 15 species. Of the four species that did not show a significant relationship, three (*Perameles bougainville*, *Petrogale lateralis*, *Setonix brachyurus*) show a strong negative trend despite the low number of populations sampled (5, 7 and 5, respectively), while the study undertaken on *Isodon obesulus* only reported six regional pairwise *F*_ST_ values with each region consisting of lumped population samples (potentially masking the relationship between *A*_r_ and *F*_ST_).

**Table 2:** Relationship between genetic diversity and genetic uniqueness in threatened Australian mammals

				Microsatellite data					
Species	Subspecies/population	Common Name	Status^a^	Populations	Loci	*R* ^2^	*b*	*P*	Reference
*Gymnobelideus leadbeateri*	-	Leadbeater's possum	CE	6	15	0.940	−0.173	<0.001	[52]; Weeks unpubl. data
*Bettongia penicillata*	*B. p. ogilbyi*	Woylie	E	9	12	0.827	−0.015	<0.001	[53]
*Burramys parvus*	-	Mountain pygmy-possum	E	12	8	0.819	−0.114	<0.001	see Table 1
*Dasyurus hallucatus*	-	Northern quoll	E	9	6	0.907	−0.166	<0.001	see Table 1
*Dasyurus maculatus*	*D. m. gacilis*	Yarri	E	9	6	0.831	−0.073	<0.001	[26]
	*D. m. maculatus (*SE mainland population*)*	Spotted-tailed Quoll (SE)	E						
		Tiger Quoll (Q)							
	*D. m. maculatus (*Queensland population*)*		V						
*Isoodon obesulus*	*I. o. obesulus*	Southern brown bandicoot (Eastern)	E	6^b^	14	0.250	−0.03	0.312	[18]
	*I. o. nauticus*	Southern brown bandicoot (Nuyts)	V						
*Perameles bougainville*	*P. b. bougainville*	Western barred bandicoot (Shark Bay)	E	5	7	0.706	−0.149	0.075	[54]
*Perameles gunnii*	*P. g.* unnamed subspecies	Eastern barred bandicoot (mainland)	E	9	12	0.942	−0.196	<0.001	see Table 1
	*P. g. gunnii*	Eastern barred bandicoot (Tasmania)	V						
*Sarcophilus harrisii*	-	Tasmanian devil	E	5	11	0.901	−0.198	0.004	[55]
*Dasyurus geoffroii*		Western quoll	V	9	5	0.446	−0.025	0.049	[20]
*Petrogale lateralis*	*P. l. hacketti*	Recherche rock-wallaby	V	7^b^	10	0.491	−0.018	0.080	[56]
	*P. l. lateralis*	Black-flanked rock-wallaby	V						
		Warru							
	*P. l.* MacDonnell Ranges race	Black-footed rock-wallaby	V						
	*P. l.* West Kimberley race		V						
*Petrogale penicillata*	-	Brush-tailed rock-wallaby	V	14	11	0.729	−0.166	<0.001	[57]
*Phascolarctos cinereus*	*P. c*. (combined populations of Qld, NSW and ACT)	Koala	V	12^b^	6	0.665	−0.023	0.002	[58]
*Potorous tridactylus*	*P. t. tridactylus*	Long-nosed potoroo (SE mainland)	V	6	10	0.807	−0.023	0.015	[59]
*Setonix brachyurus*	-	Quokka	V	5	5	0.485	−0.054	0.191	[60]

Relationship between genetic diversity (allelic richness) and genetic uniqueness (mean pairwise *F*_ST_) inferred from published microsatellite summary data where available for terrestrial mammal species listed under the Australian EPBC Act 1999

^a^Status under the Australian EPBC Act 1999

^b^Sampling/genotyping did not cover entire range and/or all subspecies/unique populations

## Discussion

Using real datasets from five threatened Australian mammal species, we have shown that there is a strong negative relationship between population neutral genetic diversity and genetic divergence, and that random genetic drift is likely to be driving this relationship. Importantly, this relationship appears to be widespread amongst threatened species, as our examination of the Australian EPBC Act 1999 listed terrestrial mammal species showed that for species where genetic data exists, over 73 % had the same significant negative relationship. This is not surprising as populations of threatened species are typically small and isolated, and therefore prone to the erosion of genetic diversity through random genetic drift [32]. A similar relationship is also observed in endemic Australian freshwater fish species that are predisposed to population bottlenecks and founder events through droughts and floods [30, 33]. While this negative relationship is expected by theory [31], to our knowledge, this is the first time empirical data from a broad range of threatened species has highlighted the profound effects of drift in natural populations.

These findings have implications for how we manage threatened species more generally. By not considering overall levels of genetic diversity across a species, and instead focussing on the conservation of unique populations defined as subspecies, ESUs or MUs, managers may inadvertently increase the likelihood of species extinction. Relatively small reductions in genetic diversity measured through neutral markers increase the threat of population extinction, particularly in changing environments [34]. Heterozygosity is intrinsically linked to additive genetic variance, or the ability to respond to environmental change [35] and theory predicts for small populations (e.g., effective population size of 100) that have low levels of genetic variation, the time to extinction in an environment with modest change may be as few as 100 generations (Additional file 1). These effects, of course, do not take into consideration factors that are already likely operating in populations such as reduced fitness through inbreeding depression (which are more severe in stressful conditions, such as suboptimal habitat) [36, 37]. Some mammal populations and species are therefore likely on a trajectory where recovery is improbable without genetic intervention.

Our analyses highlight that managers should give priority to preserving populations that account for the greatest genetic proportion of the total gene pool for a species, thereby increasing the potential for adaptation to environmental change [37–39]. Populations that have previously been considered genetically unique (and therefore managed independently) are more likely to be in need of genetic rescue and/or restoration (e.g., *Perameles gunnii* unnamed subspecies from Victoria, Australia; the southern ESU *Burramys parvus*). Guidelines for genetic rescue/restoration have been developed elsewhere [38, 40, 41], but in many cases translocation between populations in situ represents a powerful strategy for increasing genetic diversity within and between populations of threatened species where drift processes predominate [38]. Despite some risks [38, 42], translocations for genetic reasons should be considered an option in all threatened species programs at a time of rapid environmental change where ongoing adaptation is crucial for species persistence.

The perceived presence of local adaptations in geographically isolated populations in response to selective pressures associated with different environmental conditions has been a barrier to undertaking in situ translocations in threatened species [42–45]. Local adaptation is generally strongest in large populations [46], and, at least in plants, generally absent or relatively weak in small populations [44, 46]. Similarly, random genetic drift decreases local adaptation (due to a decrease in additive genetic variance), particularly in very small populations where migration is absent [37, 47]. Our results highlight that neutral genetic variation for populations of many threatened species has been decreased because of random genetic drift. By conserving populations with low levels of neutral genetic variation and in the absence of gene flow, there is a likelihood that these small populations will have reduced potential for local adaptation if it is assumed that levels of neutral variation reflect adaptive genetic variation (Additional file 1). In some cases, drift effects will be so extreme that populations could be maladapted to their environment through the random fixation of deleterious alleles [46]. Therefore, genetic uniqueness should not necessarily be considered a barrier to undertaking genetic translocations in many threatened species.

Neutral genetic markers such as microsatellites and mitochondrial sequencing have been used prolifically for determining conservation significant populations (e.g., [14–19]). Here we have highlighted how this can place emphasis on populations that are genetically depauperate and potentially maladapted. However, our approach, which focuses on neutral variation and the effects of genetic drift and gene flow, could be used to evaluate conservation significant populations with as few as six microsatellite loci. For instance, for the observed data from all five species, only three populations found in the *B. parvus* dataset sit clearly above the 95 % prediction intervals for the regression line for *A*_r_ (Additional file 1), suggesting that these populations are more differentiated than that expected under a drift model and therefore contain unique genetic diversity (novel alleles that are likely to have arisen through mutation and increased in frequency by chance through time). These three populations are from the northern ESU and have been separated from populations in the central and southern ESUs for at least 20,000–152,000 years [22]. The southern ESU has previously been regarded as the most unique *B. parvus* population [22], but this population has the least genetic variation of all populations (Additional file 1), does not sit above the regression line and therefore is not genetically unique. Similarly, the three mainland populations of *P. gunnii* are considered a subspecies, yet have the least genetic variation and do not differ from the regression line (Additional file 1), again highlighting their lack of uniqueness. It is important to note that the entire distribution of a species would need to be sampled and genotyped to be confident that a population or several populations are unique using this methodology, and this is the case here for *B. parvus*, *P. gunnii*, *D. viverrinus* and *D. hallucatus*, but not *D. maculatus* (where no samples were genotyped from populations at the northern and southern end of their distribution).

Other methods exist which can also provide information on the uniqueness of populations (e.g., [48, 49]). Similarly, with the advent of genomic approaches in conservation [50, 51], genome-wide estimates of differentiation can now be obtained for natural populations. These incorporate both neutral and adaptive loci, providing a more accurate estimate of population uniqueness, and allowing patterns of genetic diversity across populations to be contrasted for different marker systems. Ultimately, characterising variation in genes involved in local adaptation is likely to provide a clearer understanding of the link between genetic diversity, population uniqueness and adaptive potential.

## Conclusions

We have shown that random genetic drift effects in threatened animals are widespread and lead to the erosion of neutral genetic diversity within species. This places many populations and species at a greater risk of extinction in a changing environment even if other threats can be obviated. Conservation strategies need to focus on the preservation of genetic diversity at the species level, rather than that of the population, subspecies or evolutionary significant unit, and augmented gene flow from genetically diverse populations needs to be considered as a way of increasing fitness and the adaptive potential of populations.

## Abbreviations

*A*_*r*_, allelic richness; EPBC, Environment Protection and Biodiversity Conservation; ESU, evolutionary significant unit; *H*_*e*_, expected heterozygosity; IUCN, International Union for Conservation of Nature; MU, management unit

## Additional file

Additional file 1: Additional information including microsatellite simulations exploring the relationship between genetic diversity and population-specific *F*_ST_ (Appendix S1), a figure showing results of the simulations on population-specific *F*_ST_ and genetic diversity along with regressions on observed data (Appendix S2), a table presenting observed and simulated regressions statistics for Fig. 1 and Appendix S2 (Appendix S3), a table on EPBC listed terrestrial mammal species and summary information (Appendix S4), extinction risk of populations of *P. gunnii* and *B. parvus* (Appendix S5), and a figure presenting extinction risk for populations of *P. gunnii* and *B. parvus* (Appendix S6) are available online. The authors are solely responsible for the content and functionality of these materials. Queries (other than absence of the material) should be directed to the corresponding author. (DOCX 569 kb)
