# Supplementary material for: Conservation of genetic uniqueness of populations may increase extinction likelihood of endangered species: the case of Australian mammals
Source: Front Zool. 2016 Jul 8;13:31. doi: 10.1186/s12983-016-0163-z (PMC4939060; doi:10.1186/s12983-016-0163-z)
Supplement: Additional file 1: — Additional information including microsatellite simulations exploring the relationship between genetic diversity and population-specific F ST (Appendix S1), a figure showing results of the simulations on population-specific F ST and genetic diversity along with regressions on observed data (Appendix S2), a table presenting observed and simulated regressions statistics for Fig. 1 and Appendix S2 (Appendix S3), a table on EPBC listed terrestrial mammal species and summary information (Appendix S4), extinction risk of populations of P. gunnii and B. parvus (Appendix S5), and a figure presenting extinction risk for populations of P. gunnii and B. parvus (Appendix S6) are available online. The authors are solely responsible for the content and functionality of these materials. Queries (other than absence of the material) should be directed to the corresponding author. (DOCX 569 kb) [file 12983_2016_163_MOESM1_ESM.docx]

**Appendix S1.** Microsatellite simulations

We used simulations to investigate the relationship between genetic diversity and genetic uniqueness for microsatellite datasets for each species. Simulations were undertaken in R (version 3.0.2) and loading libraries *psych* and *Rlab* (from www.r-project.org). An R script was written to randomly sample two alleles with replacement at *x* loci for *N* number of individuals from a microsatellite dataset (stored as a .csv file). The first step of the simulation created a panmictic population (of *N* = 1000, for *t* = 1) from a dataset of observed genotypes from populations for each species. A stepwise mutation rate was then implemented for each locus independently, at a rate of 1x10^-4^ per generation [1]. We implemented a one-step, symmetric model, which assumes only one step per mutation, with equal probability of increasing (+1 repeat length) or decreasing (-1 repeat length) with length determined by the repeat type for the locus (e.g. di-, tri- or tetra-nucleotide repeat). Five constant population sizes (*N* = 50, 100, 250, 500, 1000) replicated five times were then created randomly (sampling with replacement) from the panmictic population of genotypes. These were then resampled independently for *t* = 20, 50, 100, 250, 500 and 1000 non-overlapping generations to create 25 genotypic datasets for each *t*. While this is a relatively simplistic model that does not account for periods of gene flow that may occur between subpopulations (which will tend to decrease divergence), it presents an upper limit to what is expected under drift and mutation. Genetic diversity (*H*_e_ and *A*_r_) and mean population-specific *F*_ST_ were then calculated as above for populations at each *t*. As mutation is likely to have relatively little impact for the small population sizes and generations used in these simulations (compared with drift), we also ran the same simulation for a single population for each species of *N* = 5000 for *t* = 5000 to determine the effects of mutation and drift. Genetic diversity (*H*_e_ and *A*_r_) and mean population-specific *F*_ST_ were then calculated as above. Prediction intervals (95%) around regression lines were calculated in R using the *predict* function.

For each species, a consistent and highly significant negative relationship (*R*^2^ > 0.95 in all cases) was found for the simulated datasets between population-specific *F*_ST_ and genetic diversity that was a quadratic relationship (Figure 2; Supporting Information). The regression equations for the observed datasets were either linear or quadratic compared with only quadratic relationships for the simulated datasets, but this likely reflects the lower number of points and narrower range of values in the actual observed datasets. When observed data were superimposed on these simulated datasets, there was a strikingly similar pattern (Appendix S2). Therefore, the strongly negative relationship found in observed data for each species and shape of this relationship is likely to be largely explained by drift processes.

**Appendix S2.** Simulations on population-specific *F*_ST_ and genetic diversity along with regressions on observed data. Regressions are based on microsatellite data from five different threatened Australian mammal species, and are between population-specific *F*_ST_ and mean allelic richness (a) or mean heterozygosity (b) for simulated (grey diamonds) and observed (black circles) data. The blue square is a single population simulation for *n* = 5000 and *t* = 5000. Best-fit regression lines are shown for simulations with 95% prediction intervals.

**Appendix S3.** Observed and simulated regressions based on microsatellite data for mean allelic diversity (*A*_r_) and mean heterozygosity (*H*_e_) against the mean population-specific *F*_ST_ for each species. Curves of best fit are shown in each case.

|  |  | Observed | | | | Simulated | | | |
| --- | --- | --- | --- | --- | --- | --- | --- | --- | --- |
| Species | Genetic diversity | *R*^2^ | *F* | *P* value | Equation | *R*^2^ | *F* | *P* value | Equation |
| *Burramys parvus* | *A*_r_ | 0.819 | 45.4 | < 0.001 | y = -0.114x + 0.861 | 0.995 | 15865 | < 0.001 | y=0.0176x^2^ - 0.295x + 1.261 |
|  | *H*_e_ | 0.809 | 42.4 | < 0.001 | y=-1.124x + 0.862 | 0.967 | 2168 | < 0.001 | y=-1.175x^2^ - 0.435x + 0.959 |
| *Perameles gunnii* | *A*_r_ | 0.988 | 243.3 | < 0.001 | y=0.093x^2^ - 0.865x + 2.184 | 0.996 | 12028 | < 0.001 | y=0.254x^2^ - 0.368x + 1.348 |
|  | *H*_e_ | 0.865 | 44.7 | < 0.001 | y=-1.724x + 1.266 | 0.982 | 3976 | < 0.001 | y=-1.186x^2^ - 0.479x + 0.968 |
| *Dasyurus viverrinus* | *A*_r_ | 0.990 | 351.7 | < 0.001 | y=0.055x^2^-0.555x + 1.411 | 0.971 | 2493 | < 0.001 | y=0.053x^2^-0.594x + 1.539 |
|  | *H*_e_ | 0.669 | 16.2 | 0.004 | y=-1.403x + 0.874 | 0.936 | 1066 | < 0.001 | y=-1.218x^2^-1.129x + 0.963 |
| *Dasyurus hallucatus* | *A*_r_ | 0.994 | 319.3 | < 0.001 | y=0.031x^2^-0.399x + 1.317 | 0.986 | 5333 | < 0.001 | y=0.024x^2^ - 0.351x + 1.306 |
|  | *H*_e_ | 0.976 | 80.9 | < 0.001 | y=-1.178x^2^-0.420x + 0.871 | 0.935 | 1051 | < 0.001 | y=-1.049x^2^-0.719x + 0.958 |
| *Dasyurus maculatus* | *A*_r_ | 0.978 | 206.7 | < 0.001 | y=0.045x^2^ - 0.519x + 1.558 | 0.986 | 5367 | < 0.001 | y=0.025x^2^-0.372x + 1.342 |
|  | *H*_e_ | 0.710 | 24.6 | < 0.001 | y=-1.579x + 1.092 | 0.970 | 2366 | < 0.001 | y=-1.248x^2^-0.474x + 0.954 |

**Appendix S4.** Terrestrial mammal species/subspecies listed under the Australian Environment Protection and Biodiversity Conservation (EPBC) Act 1999, status of populations (fragmented, declining), availability of mitochondrial or nuclear (microsatellite) genetic data in literature, and references for genetic data/fragmentation.

| EPBC status | Species/subspecies | Common name | Fragmented populations^1^ | Declining^1^ | mtDNA | Microsatellites | Reference^2^ |
| --- | --- | --- | --- | --- | --- | --- | --- |
| CE | *Gymnobelideus leadbeateri* | Leadbeater's Possum | Yes | >50% | Yes | Yes | [2] |
| CE | *Miniopterus schreibersii bassanii* | Southern Bent-wing Bat | Yes | Yes | Yes | No | [3] |
| CE | *Potorous gilbertii* | Gilbert's Potoroo | No^3^ | <100 | No | No | [4] |
| CE | *Pteropus melanotus natalis* | Christmas Island Flying-fox | No | >30% | No | No | - |
| CE | *Saccolaimus saccolaimus nudicluniatus* | Bare-rumped Sheath-tailed Bat | Yes | ~10% | No | No | [5] |
| E | *Bettongia penicillata ogilbyi* | Woylie | Yes | >90% | Yes | Yes | [6, 7] |
| E | *Bettongia tropica* | Northern Bettong | Yes | Yes | Yes | Yes | [8] |
| E | *Burramys parvus* | Mountain Pygmy-possum | Yes | Yes | Yes | Yes | [9] |
| E | *Dasyurus hallucatus* | Northern Quoll | Yes | >50% | Yes | Yes | [10] |
| E | *Dasyurus maculatus gracilis* | Spotted-tailed Quoll or Yarri (North Queensland subspecies) | Yes | <30% | Yes | Yes | [11] |
| E | *Dasyurus maculatus maculatus* | Spot-tailed Quoll, Spotted-tail Quoll, Tiger Quoll (southeastern mainland population) | Yes | <30% | Yes | Yes | [11] |
| E | *Hipposideros semoni* | Semon's Leaf-nosed Bat, Greater Wart-nosed Horseshoe-bat | Unknown | Yes | No | No | - |
| E | *Isoodon obesulus obesulus* | Southern Brown Bandicoot (Eastern) | Yes | <30% | Yes | Yes | [12, 13] |
| E | *Lagorchestes hirsutus* unnamed subsp. | Mala, Rufous Hare-Wallaby (central mainland form) | Yes^5^ | <250 | Yes | Yes | [14] |
| E | *Lasiorhinus krefftii* | Northern Hairy-nosed Wombat, Yaminon | No^3^ | ~200 | Yes | Yes | [15] |
| E | *Melomys rubicola* | Bramble Cay Melomys (rat species) | No^3^ | Yes^6^ | No | No | - |
| E | *Notoryctes caurinus* | Kakarratul, Northern Marsupial Mole | Unknown | Unknown | No | No | - |
| E | *Notoryctes typhlops* | Itjaritjari, Southern Marsupial Mole, Yitjarritjarri | Unknown | No | No | No | - |
| E | *Onychogalea fraenata* | Bridled Nail-tail Wallaby | Yes^4^ | No | Yes | Yes | [16] |
| E | *Parantechinus apicalis* | Dibbler | Yes | Yes | Yes | Yes | [17] |
| E | *Perameles bougainville bougainville* | Western Barred Bandicoot (Shark Bay) | Yes | Yes | Yes | Yes | [18] |
| E | *Perameles gunnii* unnamed subsp. | Eastern Barred Bandicoot (Mainland) | Yes^5^ | No | Yes | Yes | [19] |
| E | *Petaurus gracilis* | Mahogany Glider | Yes | Yes | No | No | - |
| E | *Petrogale persephone* | Proserpine Rock-wallaby | Yes | Yes | No | No | [20] |
| E | *Phascogale calura* | Red-tailed Phascogale | Yes | No | No | No | [21] |
| E | *Potorous longipes* | Long-footed Potoroo | Yes | Yes | No | No | [22] |
| E | *Pseudomys fumeus* | Konoom, Smoky Mouse | Yes | >30% | No | No | - |
| E | *Pseudomys oralis* | Hastings River Mouse, Koontoo | Yes | Yes | Yes | No | [23] |
| E | *Rhinolophus philippinensis* | Greater Large-eared Horseshoe Bat | Unknown | Unknown | No | No | - |
| E | *Sarcophilus harrisii* | Tasmanian Devil | Yes | 50-80% | Yes | Yes | [24] |
| E | *Sminthopsis aitkeni* | Kangaroo Island Dunnart | Unknown | Yes | No | No | - |
| E | *Sminthopsis douglasi* | Julia Creek Dunnart | Yes | Yes | No | No | - |
| E | *Sminthopsis psammophila* | Sandhill Dunnart | Yes | Yes | No | No | - |
| E | *Zyzomys palatalis* | Carpentarian Rock-rat, Aywalirroomoo | Unknown | Yes | No | No | - |
| E | *Zyzomys pedunculatus* | Central Rock-rat, Antina | Unknown | >80% | No | No | - |
| V | *Bettongia lesueur lesueur* | Burrowing Bettong (Shark Bay), Boodie | Yes | Yes | No | No | - |
| V | *Bettongia lesueur* unnamed subsp. | Burrowing Bettong (Barrow and Boodie Islands), Boodie | Yes^4^ | Yes | No | No | - |
| V | *Chalinolobus dwyeri* | Large-eared Pied Bat, Large Pied Bat | Yes | Yes | No | No | - |
| V | *Conilurus penicillatus* | Brush-tailed Rabbit-rat, Brush-tailed Tree-rat, Pakooma | Yes | >30% | No | No | - |
| V | *Dasycercus cristicauda* | Crest-tailed Mulgara | Yes | Yes | No | No | - |
| V | *Dasyuroides byrnei* | Kowari, brushy-tailed marsupial rat, Byrne's crest-tailed marsupial rat | Yes | Yes | No | No | - |
| V | *Dasyurus geoffroii* | Chuditch, Western Quoll | Yes | Yes | Yes | Yes | [25] |
| V | *Dasyurus maculatus maculatus* | Spotted-tail Quoll, Spot-tailed Quoll, Tiger Quoll (Tasmanian population) | Yes | <30% | Yes | Yes | [11, 26] |
| V | *Isoodon auratus auratus* | Golden Bandicoot (mainland) | Yes | Yes | Yes | Yes | [27] |
| V | *Isoodon auratus barrowensis* | Golden Bandicoot (Barrow Island) | Yes | Yes | Yes | Yes | [27] |
| V | *Isoodon obesulus nauticus* | Southern Brown Bandicoot (Nuyts Archipelago) | Yes | <30% | Yes | Yes | [12, 13] |
| V | *Lagorchestes conspicillatus conspicillatus* | Spectacled Hare-wallaby (Barrow Island) | Yes^4^ | Yes | No | No | - |
| V | *Lagorchestes hirsutus bernieri* | Rufous Hare-wallaby (Bernier Island) | No^3^ | Yes | No | Yes | [14] |
| V | *Lagorchestes hirsutus dorreae* | Rufous Hare-wallaby (Dorre Island) | No^3^ | Yes | No | Yes | [14] |
| V | *Lagostrophus fasciatus fasciatus* | Banded Hare-wallaby, Merrnine, Marnine, Munning | Yes | Yes | No | No | - |
| V | *Leporillus conditor* | Wopilkara, Greater Stick-nest Rat | Yes^4^ | Yes | No | No | [28] |
| V | *Macropus robustus isabellinus* | Barrow Island Wallaroo, Barrow Island Euro | No^3^ | No | Yes | Yes | [29] |
| V | *Macrotis lagotis* | Greater Bilby | Yes | >10% | Yes | Yes | [30] |
| V | *Mesembriomys macrurus* | Golden-backed Tree-rat, Koorrawal | Yes | Yes | No | No | - |
| V | *Myrmecobius fasciatus* | Numbat | Yes | Yes | Yes | No | [31] |
| V | *Notomys aquilo* | Northern Hopping-mouse, Woorrentinta | Yes | >30% | No | No | - |
| V | *Notomys fuscus* | Dusky Hopping-mouse, Wilkiniti | Yes | Yes | No | No | - |
| V | *Nyctophilus corbeni* | South-eastern Long-eared Bat | Yes | >30% | No | No | - |
| V | *Perameles gunnii gunnii* | Eastern Barred Bandicoot (Tasmania) | Yes | 30% | Yes | Yes | [19] |
| V | *Petaurus australis* unnamed subsp. | Yellow-bellied Glider (Wet Tropics), Fluffy Glider | Yes | Yes | Yes | No | [32] |
| V | *Petrogale lateralis hacketti* | Recherche Rock-wallaby | Yes | Yes | Yes | Yes | [33, 34] |
| V | *Petrogale lateralis lateralis* | Black-flanked Rock-wallaby | Yes | Yes | Yes | Yes | [33, 34] |
| V | *Petrogale lateralis* | Warru, Black-footed Rock-wallaby (MacDonnell Ranges race) | Yes | Yes | Yes | Yes | [33, 34] |
| V | *Petrogale lateralis* | Black-footed Rock-wallaby (West Kimberley race) | Yes | Yes | Yes | Yes | [33, 34] |
| V | *Petrogale penicillata* | Brush-tailed Rock-wallaby | Yes | >30% | Yes | Yes | [35] |
| V | *Petrogale xanthopus xanthopus* | Yellow-footed Rock-wallaby (SA and NSW) | Yes | Yes | Yes | Yes | [36] |
| V | *Phascogale pirata* | Northern Brush-tailed Phascogale | Yes | >30% | No | No | - |
| V | *Phascolarctos cinereus* | Koala (combined populations of Queensland, New South Wales and the Australian Capital Territory) | Yes | ~30% | Yes | Yes | [37, 38] |
| V | *Potorous tridactylus tridactylus* | Long-nosed Potoroo (SE mainland) | Yes | >30% | Yes | Yes | [39, 40] |
| V | *Pseudantechinus mimulus* | Carpentarian Antechinus | Yes | Yes | No | No | - |
| V | *Pseudocheirus occidentalis* | Western Ringtail Possum, Ngwayir | Yes | >80% | No | No | [41] |
| V | *Pseudomys australis* | Plains Rat, Palyoora | Yes | Yes | No | No | [42] |
| V | *Pseudomys fieldi* | Shark Bay Mouse, Djoongari, Alice Springs Mouse | Yes^4^ | Yes | No | No | - |
| V | *Pseudomys novaehollandiae* | New Holland Mouse, Pookila | Yes | Yes | Yes | Yes | Weeks unpubl. data |
| V | *Pseudomys pilligaensis* | Pilliga Mouse, Poolkoo | No^3^ | Yes | No | No | - |
| V | *Pseudomys shortridgei* | Dayang, Heath Rat | Yes | Yes | Yes | Yes | [43] |
| V | *Pteropus conspicillatus* | Spectacled Flying-fox | Yes | Yes | No | Yes | - |
| V | *Pteropus poliocephalus* | Grey-headed Flying-fox | No | ~30% | No | No | [44] |
| V | *Rhinonicteris aurantia* | Pilbara Leaf-nosed Bat | Yes | ~30% | Yes | No | [45] |
| V | *Setonix brachyurus* | Quokka | Yes | Yes | Yes | Yes | [46] |
| V | *Sminthopsis butleri* | Butler's Dunnart | Yes | Yes | No | No | - |
| V | *Vombatus ursinus ursinus* | Common Wombat (Bass Strait) | No^3^ | No | No | No | - |
| V | *Xeromys myoides* | Water Mouse, False Water Rat, Yirrkoo | Yes | Yes | Yes | Yes | [47] |
| V | *Zyzomys maini* | Arnhem Rock-rat, Arnhem Land Rock-rat, Kodjperr | Yes | Yes | No | No | - |

^1^Evidence for fragmentation and decline from Woinarski et al. [48].

^2^References that present genetic data on the species

^3^Species consists of a single population

^4^Species consists of a single remnant population and reintroduced population(s)

^5^No remnant populations, only reintroduced

^6^Could be extinct

**Appendix S5. Simulated extinction risk of populations of *Burramys parvus* and *Perameles gunnii***

Here we consider what the implications of our results presented in the main text are for extinction risk under a changing environment for threatened species. We consider the case of *B. parvus* and *P. gunnii*, assume that a population is being established for conservation purposes, and that the population is started with different amounts of genetic variation provided from the differentiated populations. We use simulations [48] to determine the extinction risk under a changing environment for a *B. parvus* and *P. gunnii* population with varying levels of genetic variation. These stochastic simulations assume stabilizing selection acts on a normally distributed trait which then evolves to keep up with a modest rate of environmental change (*k* = 0.1; [49]) based on a framework developed by Lynch and Lande [50]. We also assume that the new population is maintained at an effective size of 100 or 1000.

The extinction risk under different levels of genetic variation is given in Appendix S4 under a growth rate of 1 and 0.2 respectively based on simulations in Willi and Hoffmann [49], building on a previous model [50]. These indicate that the time to extinction for a small population (*N* = 100) with an intermediate level of genetic variation and growth rate of 1 is 150 generations or less, as opposed to more than 350 at the maximum level of genetic variation. When the population size is 1000, but the growth rate is only 0.2, low levels of genetic variation still result in extinction in less than 100 generations, whereas at high levels of variation populations are expected to last no more than 250 generations. In reality, empirical data suggest that the size of these populations may be reduced much further by low levels of genetic variation due to other factors like loss of fitness. For instance, recently a decrease of 70% in census size after a few generations was found when heterozygosity was decreased by 30% in the estuarine crustacean *Americamysis bahia* [51]. With effects of this magnitude, extinction risks are expected to be increased substantially.

**Appendix S6.** Relationship between genetic diversity (heterozygosity) and extinction risk for populations of (a) *Burramys parvus*, and (b) *Perameles gunnii*, under two different population sizes (*N*) and growth rates (GR) (taken from simulations in Hoffmann and Willi, [49]).

**References**

1. Dallas JF. Estimation of microsatellite mutation-rates in recombinant inbred strains of mouse. Mamm Genome. 1992;3:452-56.

2. Hansen BD, Harley DKP, Lindenmayer DB, Taylor AC. Population genetic analysis reveals a long-term decline of a threatened endemic Australian marsupial. Mol Ecol. 2009;18:3346-62.

3. Cardinal BR, Christidis L. Mitochondrial DNA and morphology reveal three geographically distinct lineages of the large bentwing bat (*Miniopterus schreibersii*) in Australia. Aust J Zool. 2000;48:1-19.

4. Sinclair EA, Costello B, Courtenay JM, Crandall KA. Detecting a genetic bottleneck in Gilbert's Potoroo (*Potorous gilbertii*) (Marsupialia: Potoroidae), inferred from microsatellite and mitochondrial DNA sequence data. Conserv Genet. 2002;3:191-6.

5. Milne DJ, Jackling FC, Sidhu M, Appleton BR. Shedding new light on old species identifications: morphological and genetic evidence suggest a need for conservation status review of the critically endangered bat, *Saccolaimus saccolaimus*. Wild Res. 2009;36:496-508.

6. Pacioni C, Wayne AF, Spencer PBS. Effects of habitat fragmentation on population structure and long-distance gene flow in an endangered marsupial: the woylie. J Zool. 2011;283:98-107.

7. Pacioni C, Wayne AF, Spencer PBS. Genetic outcomes from the translocations of the critically endangered woylie. Curr Zool. 2013;59:294-310.

8. Pope LC, Estoup A, Moritz C. Phylogeography and population structure of an ecotonal marsupial, Bettongia tropica, determined using mtDNA and microsatellites. Mol Ecol. 2000;9:2041-53.

9. Mitrovski P, Heinze DA, Broome L, Hoffmann AA, Weeks AR. High levels of variation despite genetic fragmentation in populations of the endangered mountain pygmy-possum, *Burramys parvus*, in alpine Australia. Mol Ecol. 2007;16:75-87.

10. Cardoso MJ, Eldridge MDB, Oakwood M, Rankmore B, Sherwin WB, Firestone KB. Effects of founder events on the genetic variation of translocated island populations: implications for conservation management of the northern quoll. Conserv Genet. 2009;10:1719-33.

11. Firestone KB, Elphinstone MS, Sherwin WB, Houlden BA. Phylogeographical population structure of tiger quolls *Dasyurus maculatus* (Dasyuridae: Marsupialia), an endangered carnivorous marsupial. Mol Ecol. 1999;8:1613-25.

12. Li Y, Lancaster ML, Carthew SM, Packer JG, Cooper SJB. Delineation of conservation units in an endangered marsupial, the southern brown bandicoot (*Isoodon obesulus obesulus*), in South Australia/western Victoria, Australia. Aust J Zool. 2014;62:345-59.

13. Zenger KR, Eldridge MDB, Johnston PG. Phylogenetics, population structure and genetic diversity of the endangered southern brown bandicoot (Isodon obesulus) in south-eastern Australia. Conserv Genet. 2005;6:193-204.

14. Eldridge MDB, Kinnear JE, Zenger KR, McKenzie LM, Spencer PBS. Genetic diversity in remnant mainland and "pristine" island populations of three endemic Australian macropodids (Marsupialia): *Macropus eugenii*, *Lagorchestes hirsutus* and *Petrogale lateralis*. Conserv Genet. 2004;5:325-38.

15. Banks SC, Hoyle SD, Horsup A, Sunnucks P, Taylor AC. Demographic monitoring of an entire species (the northen hairy-nosed wombat, *Lasiorhinus krefftii*) by genetic analysis of non-invasively collected material. Anim Conserv. 2003;6:101-7.

16. Sigg DS. Reduced genetic diversity and significant genetic differentiation after translocation: comparison of the remnant and translocated populations of bridled nailtail wallabies (*Onychogalea fraenata*). Conserv Genet. 2006;7:577-89.

17. Mills HR, Moro D, Spencer PBS. Conservation significance of island versus mainland populations: a case study of dibblers (*Paratechinus apicalis*) in Western Australia. Anim Conserv. 2004;7:387-95.

18. Smith S, Hughes J. Microsatellite and mitochondrial DNA variation defines island genetic reservoirs for reintroductions of an endangered Australian marsupial, *Perameles bougainville*. Conserv Genet. 2008;9:547-57.

19. Weeks AR, van Rooyen A, Mitrovski P, Heinze D, Winnard A, Miller AD. A species in decline: genetic diversity and conservation of the Victorian eastern barred bandicoot, *Perameles gunnii*. Conserv Genet. 2013;14:1243-54.

20. Potter S, Cooper SJB, Metcalfe CJ, Taggart DA, Eldridge MDB. Phylogenetic relationships of rock-wallabies, *Petrogale* (Marsupialia: Macropodidae) and their biogeographic history within Australia. Mol Phylogenet Evol. 2012;62:640-52.

21. Spencer PBS, Rhind SG, Eldridge MDB. Phylogenetic structure within *Phascogale* (Marsupialia: Dasyuridae) based on partial cytochrome *b* sequence. Aust J Zool. 2001;49:369-77.

22. Luikart G, Painter J, Crozier RH, Westerman M, Sherwin WB. Characterization of microsatellite loci in the endangered long-footed potoroo *Potorous longipes*. Mol Ecol. 1997;6:497-8.

23. Rowe KMC, Rowe KC, Elphinstone MS, Baverstock PR. Population structure, timing of divergence and contact between lineages in the endangered Hastings River mouse (*Pseudomys oralis*). Aust J Zool. 2011;59:186-200.

24. Jones ME, Paetkau D, Geffen E, Moritz C. Genetic diversity and population structure of Tasmanian devils, the largest marsupial carnivore. Mol Ecol. 2004;13:2197-209.

25. Cardoso MJ: Conservation genetics of Australian quolls. University of New South Wales, Biological, Earth and Environmental Sciences; 2011.

26. Firestone KB, Houlden BA, Sherwin WB, Geffen E. Variability and differentation of microsatellites in the genus *Dasyurus* and conservation implications for the large Australian carnivorous marsupials. Conserv Genet. 2000;1:115-33.

27. Ottewell K, Dunlop J, Thomas N, Morris K, Coates D, Byrne M. Evaluating success of translocations in maintaining genetic diversity in a threatened mammal. Biol Conserv. 2014;171:209-19.

28. Barclay SD, Costello B, Sherwin WB. Limited cross-species microsatellite amplification and the isolation and characterization of new microsatellite markers for the greater stick-nest rat (*Leporillus conditor*). Mol Ecol Notes. 2006;6:882-5.

29. Eldridge MDB, Potter S, Johnson CN, Ritchie EG. Differing impact of a major biogeographic barrier on genetic structure in two large kangaroos from the monsoon tropics of Northern Australia. Ecol Evol. 2014;4(5):554-67.

30. Moritz C, Heideman A, Geffen E, McRae P. Genetic population structure of the greater bilby *Macrotis lagotis*, a marsupial in decline. Mol Ecol. 1997;6:925-36.

31. Fumagalli L, Moritz C, Taberlet P, Friend JA. Mitochondrial DNA sequence variation within the remnant populations of the endangered numbat (Marsupialia: Myrmecobiidae: *Myrmecobius fasciatus*). Mol Ecol. 1999;8:1545-9.

32. Meredeth B, Cooksley H, Carthew SM, Cooper SJB. Conservation units and phylogeographic structure of an arboreal marsupial, the yellow-bellied glider (*Petaurus australis*). Aust J Zool. 2006;54:305-17.

33. Lennon MJ, Taggart DA, Temple-Smith PD, Eldridge MDB. The impact of isolation and bottlenecks on genetic diversity in the Pearson Island population of the black-footed rock-wallaby (*Petrogale lateralis pearsoni*; Marsupialia: Macropodidae). Aust Mammal. 2011;33:152-61.

34. Ruykys L, Lancaster ML. Population structure and genetic diversity of the black-footed rock-wallaby (*Petrogale lateralis* MacDonnell Ranges race). Aust J Zool. 2015;63(2):91-100.

35. Hazlitt SL, Goldizen AW, Nicholls JA, Eldridge MDB. Three divergent lineages within an Australian marsupial (*Petrogale penicillata*) suggest multiple major refugia for mesic taxa in southeast Australia. Ecol Evol. 2014;4:1102-16.

36. Pope LC, Sharp A, Moritz C. Population structure of the yellow-footed rock-wallaby Petrogale xanthopus (Gray, 1854) inferred from mtDNA sequences and microsatellite loci. Mol Ecol. 1996;5:629-40.

37. Houlden BA, England PR, Taylor AC, Greville WD, Sherwin WB. Low genetic variability of the koala *Phascolarctos cinereus* in south-eastern Australia following a severe populations bottleneck. Mol Ecol. 1996;5:269-81.

38. Lee KE, Seddon JM, Corley SW, Ellis WAH, Johnston SD, de Villiers DL, Preece HJ, Carrick FN. Genetic variation and structuring in the threatened koala populations of Southeast Queensland. Conserv Genet. 2010;11:2091-103.

39. Frankham GJ, Handasyde KA, Eldridge MDB. Novel insights into the phylogenetic relationships of the endangered marsupial genus *Potorous*. Mol Phylogenet Evol. 2012;64:592-602.

40. Frankham GJ, Handasyde KA, Norton M, Murray A, Eldridge MDB. Molecular detection of intra-population structure in a threatened potoroid, *Potorous tridactylus*: conservation management and sampling implications. Conserv Genet. 2014;15:547-60.

41. Wilson K, de Tores P, Spencer PBS. Isolation and characterisation of polymorphic microsatellite markers in the western ringtail possum, *Pseudocheirus occidentalis*. Conserv Genet Resour. 2009;1:123-5.

42. Brandle R, Moseby KE, Adams M. The distribution, habitat requirements and conservation status of the plains rat, *Pseudomys australis* (Rodentia: Muridae). Wildlife Research. 1999;26:463-77.

43. Salinas M, Bunce M, Cancilla D, Alpers DL, Spencer PBS. Divergent lineages in the heath mouse (*Pseudomys shortridgei*) are indicative of major contraction to geogrpahically isolated refugia on the eastern and western sides of Australia during the early Pleistocene. Aust J Zool. 2009;57:41-7.

44. Webb NJ, Tidemann CR. Mobility of Australian flying-foxes, *Pteropus* spp. (Megachiroptera): evidence from genetic variation. Proc Roy Soc Lond B. 1996;263:497-502.

45. Armstrong KN. Phylgeographic structure in *Rhinonicteris aurantia* (Chiroptera: Hipposideridae): implications for conservation. Acta Chiropterol. 2006;8:63-81.

46. Alacs EA, Spencer PBS, de Tores PJ, Krauss SL. Population genetic structure of island and mainland populations of the quokka, *Setonix brachyurus* (Macropodidae): a comparison of AFLP and microsatellite markers. Conserv Genet. 2011;12:297-309.

47. Benfer D, Baker AM, Ball T, Gynther I, Janetzki H, Fuller S. Conservation genetics of the water mouse, *Xeromys myoides* Thomas, 1889. Aust J Zool. 2014;62:382-92.

48. Woinarski JCZ, Burbidge AA, Harrison PL: *The Action Plan for Australian Mammals 2012.* Melbourne: CSIRO Publishing; 2014.

49. Willi Y, Hoffmann AA. Demographic factors and genetic variation influence population persistence under environmental change. J Evol Biol. 2009;22:124-33.

50. Lynch M, Lande R: Evolution and extinction in response to environmental change. In *Biotic interactions and global change.* Edited by Kareiva PM, Kingsolver JG, Huey RB. Sunderland, MA: Sinauer Associates; 1993:234-50.

51. Markert JA, Champlin DM, Gutjahr-Gobeli R, Grear JS, Kuhn A, McGreevy TJ, Roth A, Bagley MJ, Nacci DE. Population genetic diversity and fitness in multiple environments**.** BMC Evol Biol. 2010;10.
